# SHLP6: a novel NLRP3 and Cav1 modulating agent in Cu-induced oxidative stress and neurodegeneration

**DOI:** 10.3389/fnmol.2025.1553308

**Published:** 2025-04-01

**Authors:** H. Thamarai Kannan, Suganiya Umapathy, Ieshita Pan

**Affiliations:** Department of Medical Biotechnology, Saveetha School of Engineering, Institute of Biotechnology, Saveetha Institute of Medical and Technical Sciences, Chennai, Tamil Nadu, India

**Keywords:** zebrafish larvae, SHLP6, CuSO_4_, oxidative stress, inflammation, toxicity

## Abstract

**Introduction:**

Copper sulfate exposure induces oxidative stress by triggering excessive reactive oxygen species (ROS) production, leading to inflammatory responses, neuroinflammation, and cellular dysfunction. Small humanin-like peptide-6 (SHLP-6), a mitochondria-derived peptide with anti-aging and anti-cancer properties, has not been explored for its protective effects against copper sulfate toxicity. This study investigates the antioxidant, anti-inflammatory, and neuroprotective potential of SHLP-6 in zebrafish larvae exposed to copper sulfate.

**Methods:**

Zebrafish larvae were exposed to copper sulfate and treated with SHLP-6 at concentrations ranging from 10 to 50 μg/mL. ROS-scavenging activity was assessed using *in vitro* assays, and enzymatic antioxidant markers, lipid peroxidation, nitric oxide levels, acetylcholine esterase (AChE) activity, and locomotor behavior were evaluated. Additionally, gene expression analysis was performed for inflammatory and antioxidant markers.

**Results:**

Treatment with SHLP-6 at 40 μg/mL significantly reduced malformations, improved heart rate (178 bpm), and increased survival rates (85%) in zebrafish larvae. The highest ROS inhibition was observed at 58.7% and 74.3%, while antioxidant enzyme activity was enhanced, with superoxide dismutase (68.3 U/mg), catalase (82.40 U/mg), and reduced glutathione (79.3 U/mg). Lipid peroxidation and nitric oxide levels decreased to 3.86 and 3.41 U/mg, respectively. SHLP-6 improved AChE levels (78.3 U/mg) and locomotor activity (43.53 m distance travelled).

**Discussion:**

SHLP-6 upregulated TNF-α (2.16-fold), NLRP3 (1.78-fold), and COX-2 (0.705-fold), while increasing IL-10 (1.84-fold), suggesting neuroinflammation modulation. Antioxidant gene expression (SOD, CAT, GST, and GSH) was significantly upregulated. These findings indicate SHLP-6’s potential as a neuroprotective and antioxidant agent against copper sulfate-induced toxicity.

## Graphical abstract

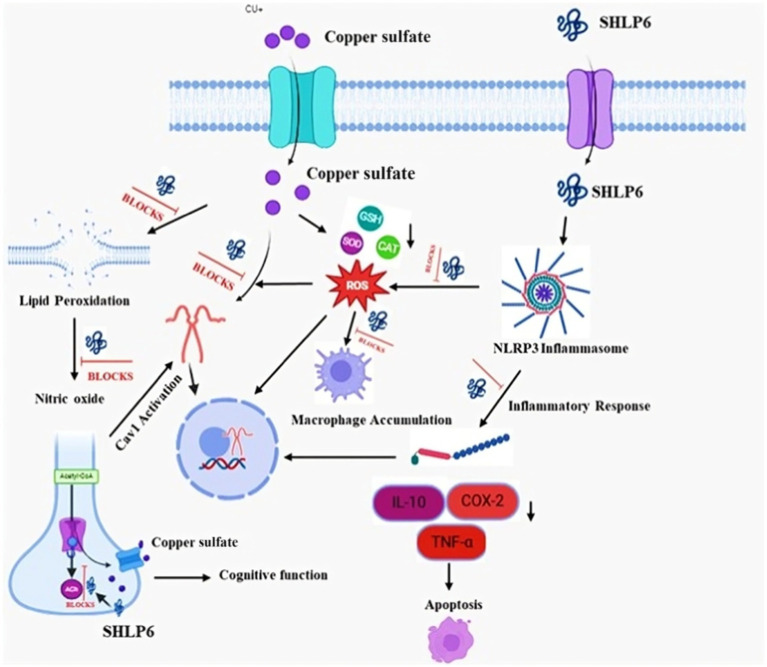

## Highlights

- SHLP6 improved the survival and heart rate in zebrafish under toxicity.
- SHLP6 mitigated Cu-induced ROS by enhancing key antioxidant enzymes (SOD, CAT, and GSH) and reduced lipid peroxidation.
- SHLP6 modulated inflammation through the regulation of TNF-α, NLRP3, IL-10, and Cox-2 expression.
- SHLP6 showed neuroprotective potential by restoring acetylcholine esterase and improving locomotion.

## Introduction

Copper is a vital micronutrient that serves as a cofactor for essential copper-dependent enzymes, but, at high concentrations, it exhibits toxicity to many cell types due to its redox cycling capabilities (Focarelli et al., 2022). Copper ions transition between the Cu^2+^ and Cu^+^ states, generating reactive oxygen species (ROS) such as superoxide anions, hydroxyl radicals, and hydrogen peroxide (Vo et al., 2024). This process leads to oxidative damage by depleting cellular glutathione (GSH), a critical antioxidant, and impairing the activities of key antioxidant enzymes, such as superoxide dismutase (SOD), catalase (CAT), and glutathione peroxidase (GPx) (Zhang et al., 2022). The resulting imbalance increases lipid peroxidation (LPO), damages proteins, disrupts mitochondrial function, and triggers apoptosis through oxidative stress pathways (Su et al., 2019). In zebrafish models, copper sulfate (CuSO_4_) exposure induces hepatic and systemic oxidative stress, as evidenced by increased LPO, altered antioxidant enzyme activity, and mitochondrial DNA damage (Singh et al., 2022). Oxidative stress occurs when there is an imbalance between cellular oxidative processes and antioxidant defense mechanisms, primarily due to the overproduction of free radicals and ROS. High levels of copper worsen this condition by triggering an excess production of ROS, which can lead to DNA damage and the oxidative breakdown of biological macromolecules (Newsholme et al., 2016). Furthermore, the gathering of immune cells in inflamed areas exacerbates tissue damage and oxidative stress. Inflamed immune cells release a variety of cytokines and chemokines that attract more immune cells to the affected site, escalating the inflammatory response and oxidative damage (Chatterjee, 2016).

Inflammation is a complex biological response triggered by harmful stimuli, such as pathogens, damaged cells, or toxic compounds (Chen et al., 2018). Inflammation can induce apoptosis, a programmed cell death mechanism, through several interconnected pathways involving immune signaling and oxidative stress (Messer, 2017). During inflammation, immune cells such as macrophages and neutrophils release pro-inflammatory cytokines (e.g., TNF-α, IL-1β) and chemokines that can activate intrinsic and extrinsic apoptotic pathways (Wright et al., 2010; Brostjan and Oehler, 2020). Inflammation-induced apoptosis is closely linked to neurodegeneration, as chronic neuroinflammation and oxidative stress are key contributors to the progression of neurodegenerative diseases (Zhang et al., 2023). In conditions such as Alzheimer’s disease, Parkinson’s disease, and multiple sclerosis, the persistent activation of microglia and astrocytes (the primary immune cells of the central nervous system) results in the release of pro-inflammatory cytokines and excessive ROS (Kwon and Koh, 2020; Zhang et al., 2023). Neuroinflammation-driven apoptosis also disrupts synaptic integrity and neuronal communication, leading to cognitive and motor impairments commonly observed in neurodegenerative diseases (Gao et al., 2023). These inflammatory mediators lead to mitochondrial dysfunction and cytokine signaling via TNF receptors, further contributing to neuronal loss (López-Armada et al., 2013; Rehman and Akash, 2016).

Several pharmacological interventions have been developed to mitigate neuroinflammation, oxidative stress, and apoptosis in neurodegenerative diseases. The goal is to slow disease progression and preserve neuronal function. These drug therapies include non-steroidal anti-inflammatory drugs (NSAIDs) such as ibuprofen and celecoxib, corticosteroids (dexamethasone and prednisone), monoclonal antibodies (aducanumab and natalizumab), antioxidants (vitamin E, coenzyme Q10, and N-acetylcysteine), and mitochondria-targeted antioxidants (MitoQ and SkQ1) (Shefrin and Goldman, 2009; Gordo et al., 2017; Jia et al., 2022; Raeeszadeh et al., 2022; Lereim et al., 2024). Additionally, neuroprotective agents, such as rasagiline and selegiline, and calcium channel blockers, such as nimodipine, have been used (Hohmann et al., 2022; Naoi et al., 2022). Despite advancements in pharmacological intervention, the majority of current therapies only provide symptomatic relief and do not effectively halt neurodegeneration.

Mitochondria play important roles in mitophagy, the generation of ROS, and calcium signaling that influence the fate of cells (Kuo et al., 2024). Small humanin-like peptide 6 (SHLP6), derived from mitochondrial small open reading frames (sORFs), plays a pivotal role in modulating inflammation by targeting mitochondrial function. Mitochondria are critical regulators of inflammatory pathways, and SHLP6 exerts its effects by maintaining mitochondrial health, mitigating oxidative stress, and regulating immune responses (Merry et al., 2020). By enhancing mitochondrial antioxidant defenses, excessive ROS production can be reduced (Shi et al., 2013). As a mitochondrial-derived peptide encoded within the 16S rRNA region, SHLP6 has dual functionality, exhibiting pro-apoptotic activity in cancer cells and cytoprotective effects in normal cells, making it suitable for targeted therapies (Li et al., 2024). The neuroprotective properties of mitochondria-derived peptides can regulate apoptotic pathways, ensuring neuronal survival under stress conditions (Merry et al., 2020). This study evaluates the effectiveness of SHLP6 in ameliorating CuSO_4_-induced oxidative stress and inflammation by lowering cellular toxicity, suppressing pro-inflammatory cytokines, and enhancing antioxidant defenses in zebrafish larval models.

## Materials and methods

### Zebrafish maintenance and embryo collection

Adult male and female zebrafish were obtained from Tarun Fish Farm in Manimangalam (latitude N 12°55′1″ and longitude E 80°2′29″), Chennai. They were kept in a 19 L glass tank at 28.5°C with a 14/10-h light/dark cycle and fed live *Artemia salina* (brine shrimp) three times per day. After 20 days of acclimation to the laboratory environment, the fish were bred in separate spawning tanks with a 1:1 male–female ratio. Spawning was initiated at the start of the light cycle. The embryos were removed from the breeding unit 30 min after the light cycle began and thoroughly washed with embryonic (E3) medium containing 0.17 mM KCl, 5 mM NaCl, 0.33 mM CaCl_2,_ 0.33 mM MgSO_4_, and 0.1% methylene blue. After 30 min, the eggs were extracted, medium-sized embryos were cleaned, and incubated at room temperature in a 12-well plate until chemical treatment. The collected embryos were inspected under a microscope to differentiate between fertilized and unfertilized embryos; fertilized embryos with normal morphology were used for subsequent research. After 4 h post-fertilization (hpf), zebrafish larvae were divided into groups: a control group (untreated), a stress group (CuSO_4_; 10 μm), and a treatment group (SHLP6; 10, 20, 30, 40, and 50 μg/mL). CuSO_4_ concentration was determined based on the previously reported studies (Hernandez et al., 2011; Singh et al., 2022). The larvae were given egg yolk throughout the experiment. On the fourth day, the larvae were homogenized and used for further experiments (Sudhakaran et al., 2022).

### *In vivo* developmental toxicity studies

#### Heart rate and survival rate

The survival and heart rate of larvae exposed to untreated control, CuSO_4_-exposed, and SHLP6-treated groups (10, 20, 30, 40, and 50 μg/mL) were evaluated. The heart rate of zebrafish larvae was measured by counting the atrial and ventricular contractions under a microscope for 1 min (Sudhakaran et al., 2022).

### *In vitro* antioxidant studies

#### DPPH assay

The DPPH assay was conducted with minor modifications (Velumani et al., 2024). SHLP6 (10, 20, 30, 40, and 50 μg/mL) and Trolox were combined and added to the DPPH solution. The reaction mixture was incubated in the dark for 30 min. After incubation, the absorbance was measured at 517 nm using an ELISA plate reader (Thermo Fisher Scientific, Waltham, Massachusetts, USA).

#### ABTS assay

The ABTS scavenging activity of SHLP6 was determined using a previously described method (Velumani et al., 2024). Briefly, ABTS (7 mM) was mixed with potassium persulfate (2.45 mM). The sample was diluted with 0.2 M PBS (pH 7.4) to achieve an absorbance of 0.70 ± 0.02. Trolox and various concentrations of SHLP6 were added to a diluted ABTS solution. After 6 min of incubation at room temperature, the absorbance was measured at 734 nm using an ELISA plate reader (Thermo Fisher Scientific, Waltham, Massachusetts, USA).

### Fluorescent staining

#### DCFDA

Zebrafish larvae were used to detect intracellular ROS formation using DCFDA staining. The DCFDA staining procedure was performed according to a previously published protocol (Singh et al., 2022). The CuSO_4_-exposed larvae and SHLP6-treated larvae (10, 20, 30, 40, and 50 μg/mL) were incubated with a DCFDA solution for 1 h at room temperature in the dark. After incubation, the larvae were anesthetized, washed with fresh E3 medium, and visualized under a Magnus Olympus fluorescence microscope. ImageJ software was used to quantify fluorescence intensity.

#### Acridine orange

Acridine orange staining dye was used to assess apoptosis. The larvae were stained with acridine orange for 1 h at room temperature in the dark. After staining, the larvae were washed and anesthetized for visualization. Fluorescence images were captured using an Olympus fluorescence microscope. ImageJ software was used to quantify fluorescence intensity (Singh et al., 2022).

#### DPPP

LPO was detected using the fluorescent stain DPPP. The larvae were stained with DPPP and incubated for 30 min in the dark. Before visualization, the larvae were anesthetized and cleaned in an embryo medium. The images of the stained larvae were then examined using an Olympus fluorescence microscope. ImageJ software was used to quantify fluorescence intensity (Singh et al., 2022).

### Macrophage accumulation

#### Neutral red

To achieve optimal macrophage staining in larvae, they were exposed to untreated control, CuSO_4_-exposed, and SHLP6-treated groups (10, 20, 30, 40, and 50 μg/mL). Larvae were treated in the dark for 3–6 h with a neutral red solution containing 2.5 μg/mL. Macrophage accumulation was observed using a Magnus compound microscope (Sudhakaran et al., 2022).

### Enzymatic assays

#### Estimation of SOD and CAT activity

SOD and CAT assays were performed as previously reported (Issac et al., 2021). A reaction mixture of 750 mM NBT, 130 mM methionine, and 20 mM riboflavin was added to the supernatant. The mixture was incubated for 20 min and quantified at 420 nm. To the 50 μL of supernatant, 100 μL of buffered H_2_O_2_ was added and kept in a water bath at 70°C for 20 min. Absorbance was measured at 240 nm for 2 min using a microplate reader (Thermo Fisher Scientific, Waltham, Massachusetts, USA).

#### Estimation of GSH activity

A 100-μL larval sample was mixed with 50 μL of 20 mM DTNB and 150 μL of 100 mM potassium phosphate buffer (pH 7.4). Absorbance was recorded after 10 min at 412 nm (Issac et al., 2021).

#### Estimation of LPO activity

Malondialdehyde levels were quantified using the thiobarbituric acid assay. A 100-μL larval homogenate was mixed with 5% trichloroacetic acid (0.1 mL) and incubated for 15 min on ice (Issac and Velumani, 2024). Subsequently, 0.67% thiobarbituric acid (0.2 mL) was added and heated in a water bath at 100°C for 30 min. The mixture was cooled on ice for 20 min and then centrifuged at 2,000 rpm for 10 min. The absorbance of the resulting supernatant was recorded at 535 nm to assess the malondialdehyde (MDA) content (Issac et al., 2021).

#### Estimation of NO assay

The Griess method was used to assess the nitric oxide (NO) levels (Schulz et al., 1999). Griess reagent (100 μL) was added to larval homogenate (100 μL), and the mixture was incubated for 25 min at room temperature. The absorbance of the resulting supernatant was recorded at 540 nm.

#### RT-PCR

Using RDP Trio™ Reagent, RNA was extracted from zebrafish larvae. Primers were designed for antioxidant, inflammatory, and neurodegenerative genes using NCBI Primer-BLAST (Table 1). AURA 2× One-Step RT-PCR Master Mix was used to investigate gene expression. The reverse transcription process was initiated with a 15-min cycle at 44–50°C, followed by a 3-min gene activation at 95°C. Denaturation was performed for 10 s at 95°C and continued for 40 cycles at 60°C. Annealing was performed for 45 s, followed by a 15-s extension at 72°C. The fold change was calculated using the 2^-ΔΔCT^ method (Sudhakaran et al., 2022).

**Table 1:** Primers for gene expression analysis.

S. no.	Gene	Forward primer (5′–3′)	Reverse primer (5′–3′)
1	β-actin	AAGCTGTGACCCACCTCACG	GGCTTTGCACATACCGGAGC
2	TNF-α	GGAGAGTTGCCTTTACCGCT	GTCTGTGCCCAGTCTGTCTC
3	IL-10	CTTTAAAGCACTCCACAACCCCAA	CTTGCATTTCACCATATCCCGCTT
4	Cox-2	GGATGATGAGAGAATCTTCCAAACC	TTCCAGAACTTTAACAGCGACTCC
5	Cav 1	AAAACTCCCCACAGAAAGCAAT	CCGTGCCTGAAGTGCGAT
6	NLRP3	TGAACAGGTTGATGACTGATATGCT	ACAGCGATTTTCCCAGCATCCTTGC
7	SOD	GGTCCGCACTTCAACCCTCA	TACCCAGGTCTCCGACGTGT
8	CAT	AACTGTGGAAGGAGGGTCGC	CGCTCTCGGTCAAAATGGGC
9	GSH	TAAACAAAACCGAAGATGGG	TTAAACCTGTAGCCGAAAGC
10	GST	TCTGGACTCTTTCCCGTCTCTCAA	ATTCACTGTTGCCGTTGCCGT

### Cognitive-impairment study

#### Acetylcholinesterase activity

Zebrafish larvae were exposed to CuSO_4_ to induce neurotoxicity after treatment with SHLP6. Larvae were anesthetized with tricaine, homogenized in PBS, and the supernatant was collected. The supernatant was then mixed with 3.3 mM 5,5′-dithiobis (2-nitrobenzoic acid) and incubated for 20 min. Acetylcholine was subsequently added, and the absorbance was measured at 412 nm using a microplate reader (Thermo Fisher Scientific, Waltham, Massachusetts, USA) (Issac and Velumani, 2024).

#### Locomotory behavior

The locomotor behavior of larvae was analyzed based on the swimming patterns. At 6 days post-fertilization (dpf), larvae (*n* = 3) from each experimental group were placed in a white ice tray filled with E3 medium (3 mL) for 15 min of acclimation. The movements of the larvae were recorded for 1 min in a quiet environment and analyzed three times. The video recordings were analyzed using the UMA Tracker software (Velumani et al., 2024).

### Statistical analysis

All experiments were conducted in triplicate and reported as mean ± SD. GraphPad Prism 5.0 (GraphPad Software, Inc., San Diego, CA) was used to perform a one-way analysis of variance (ANOVA) and Dunnett’s Multiple Comparison Test (Qamar et al., 2020). Data are denoted with “*” to indicate significance at a *p*-value of <0.05.

## Results

### Zebrafish developmental toxicity, heart rate, and survival rate

**Figure 1:**
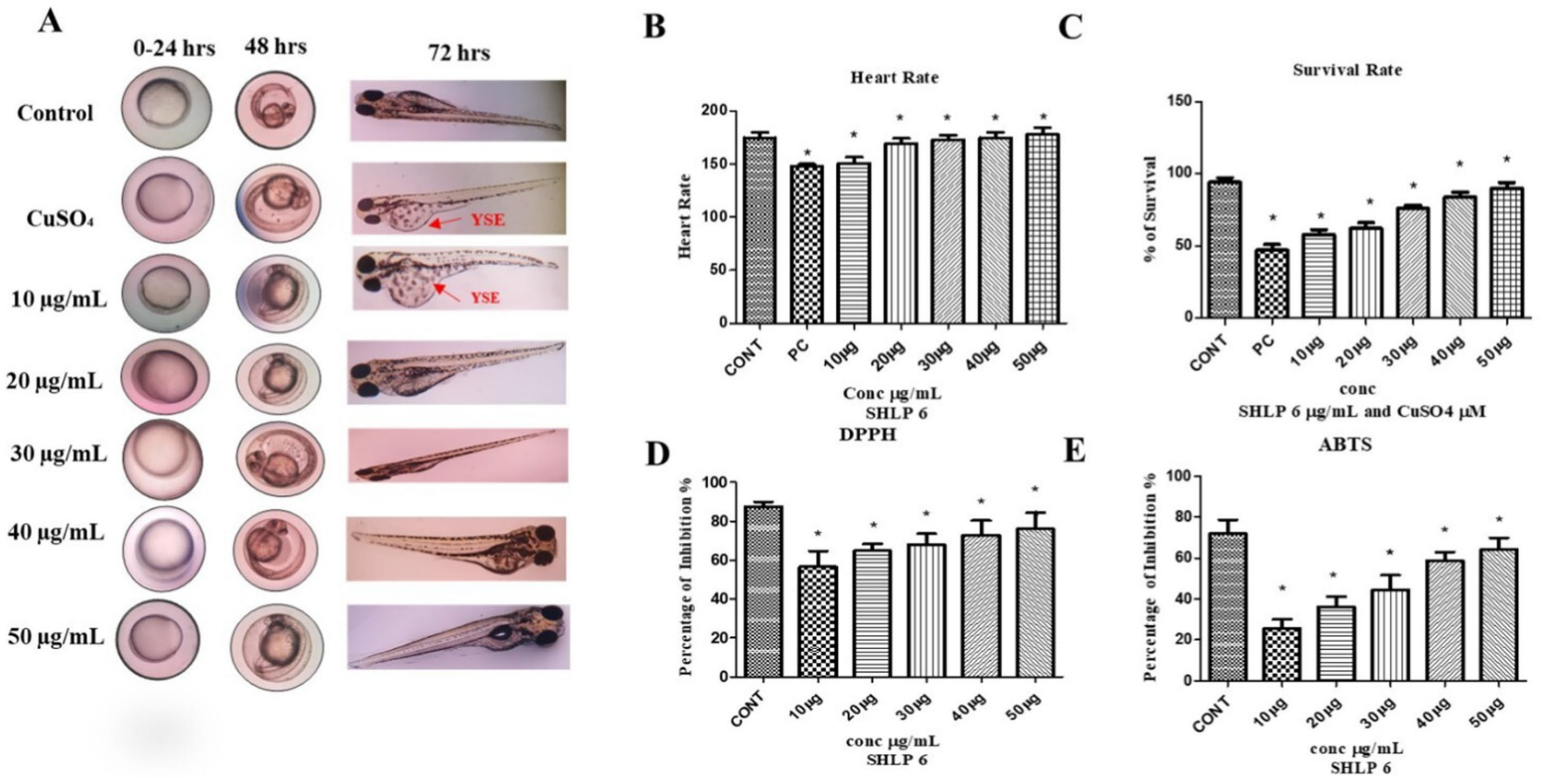
Developmental toxicity analysis of zebrafish larvae and antioxidant activity of SHLP6 (Scale bar 200 µm). **(A)** Zebrafish embryos exposed to CuSO4 were treated with SHLP6 (10 -50 µg/mL), **(B)** Heart rate, **(C)** Survival rate, **(D)** DPPH assay, and **(E)** ABTS assay. Asterisks (*) indicate statistical significance at a *p*-value of <0.05.

CuSO_4_-induced developmental toxicity was assessed in zebrafish embryos. The control group showed no aberrant morphological alterations, while the CuSO_4_-exposed group exhibited malformations such as yolk sac edema (YSE). CuSO_4_-exposed larvae treated with SHLP6 showed a reduced probability of malformation, whereas the lower concentration (10 μg/mL) was not effective against malformations such as YSE induced by CuSO_4_ stress. To investigate the cardiotoxicity of CuSO_4_, the heart rate of zebrafish larvae was measured. The results showed a significant decrease in heart rate (147 bpm) in the CuSO_4_-exposed group compared to the control group (178 bpm). However, the SHLP6-treated groups—10 μg/mL (158 bpm), 20 μg/mL (168 bpm), 30 μg/mL (172 bpm), 40 μg/mL (174 bpm), and 50 μg/mL (178 bpm)—exhibited a notable recovery of heart rate and an increase in survival rate to 85% in zebrafish larvae exposed to CuSO_4_. Additionally, the survival rate in the CuSO_4_-exposed group was 45%, whereas the survival rate in the SHLP6-treated groups increased in a dose-dependent manner at a higher concentration of 50 μg/mL (85%) (Figures 1A–C). These findings indicate that SHLP6 was effective in reducing the mortality and malformation rates.

### Antioxidant activity

The antioxidant activity of SHLP6 was assessed using DPPH and ABTS scavenging studies. Trolox was used as a positive control and showed 71.5% inhibition in DPPH and 87.4% inhibition in ABTS. SHLP6 showed concentration-dependent activity with 25.6% (10 μg/mL), 36.2% (20 μg/mL), 44.5% (30 μg/mL), 58.7% (40 μg/mL), and 64.2% (50 μg/mL), respectively. Similarly, the ABTS results showed inhibition of 56.7% (10 μg/mL), 64.85% (20 μg/mL), 68.12% (30 μg/mL), 73% (40 μg/mL), and 74.3% (50 μg/mL), respectively (*p* < 0.05) (Figures 1D,E), indicating significant antioxidant effectiveness.

### DCFDA staining

**Figure 2:**
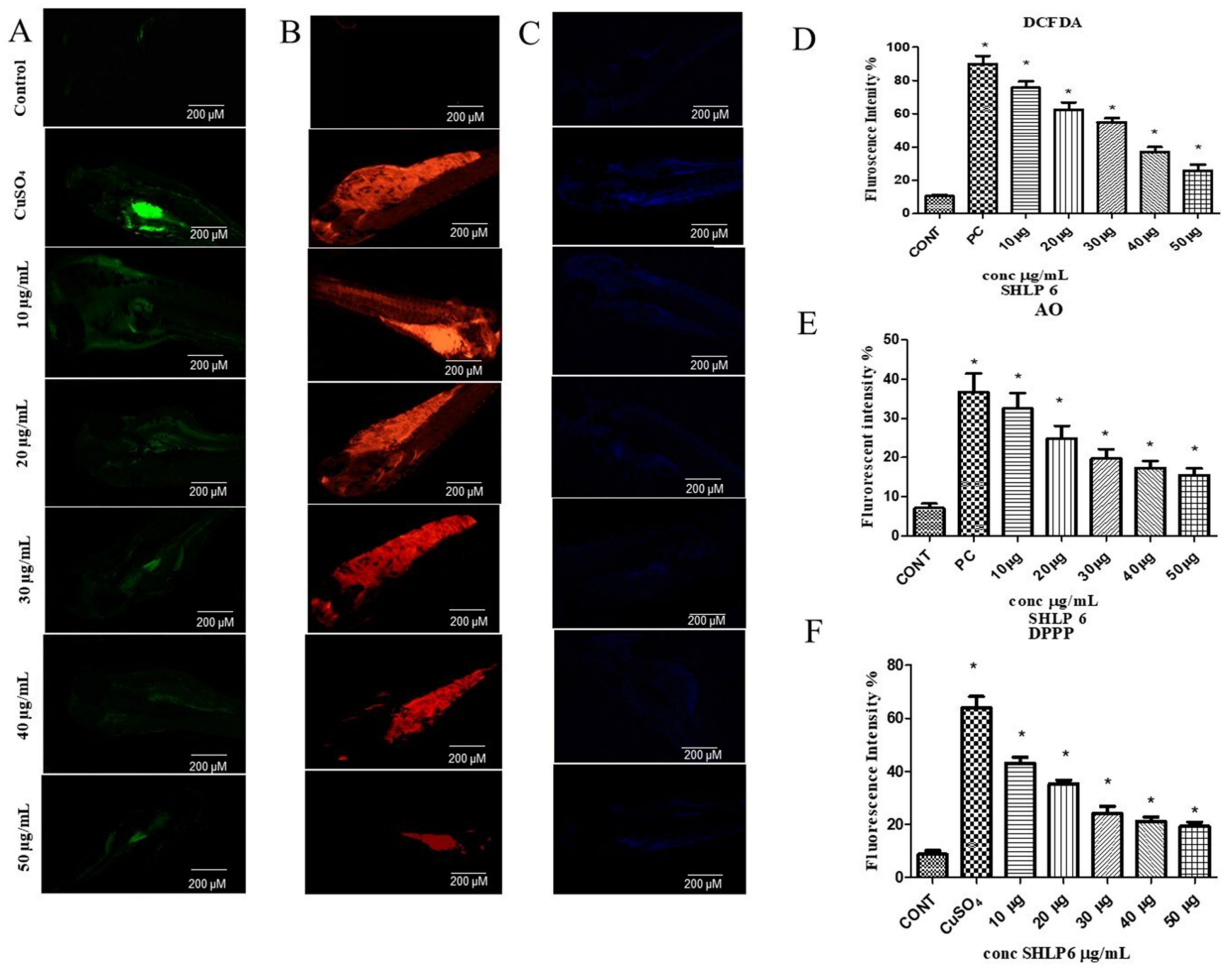
Fluorescent staining. **(A)** DCFDA staining, **(B)** AO staining, **(C)** DPPP staining. Mean fluorescence intensity, **(D)** DCFDA staining, **(E)** AO staining, **(F)** DPPP staining. Asterisks (*) indicate the statistical significance at a *p*-value of <0.05.

DCFDA was used to determine the level of ROS activity. In CuSO_4_-exposed larvae, ROS levels were higher with a fluorescence intensity of 82.3%. SHLP6 (50 μg/mL) significantly reduced ROS levels with a fluorescence intensity of 20.3% compared to the control with a fluorescence intensity of 10% (Figures 2A,D).

### AO staining

Apoptosis in zebrafish larvae was observed through AO staining. The control group showed an 8% fluorescence intensity. In contrast, the larvae exposed to CuSO_4_ exhibited a 36.6% increase in fluorescence intensity. However, when treated with SHLP6 (50 μg/mL), the apoptosis in zebrafish larvae was reduced with a fluorescence intensity of 17.6% (Figures 2B,E). SHLP6 significantly decreased apoptosis in zebrafish larvae compared to those exposed to CuSO_4_.

### DPPP staining

Using fluorescent staining with DPPP, LPO levels were determined in zebrafish larvae. The control group exhibited 8.6% fluorescence intensity. In CuSO_4_-exposed larvae, the fluorescence intensity increased to 64.02%, indicating higher LPO levels. However, in SHLP6 (50 μg/mL)-treated larvae, LPO levels were significantly reduced to 19.28% (Figures 2C,F). SHLP6 treatment significantly decreased LPO levels in zebrafish larvae.

### Effects of macrophage accumulation on zebrafish larvae

**Figure 3:**
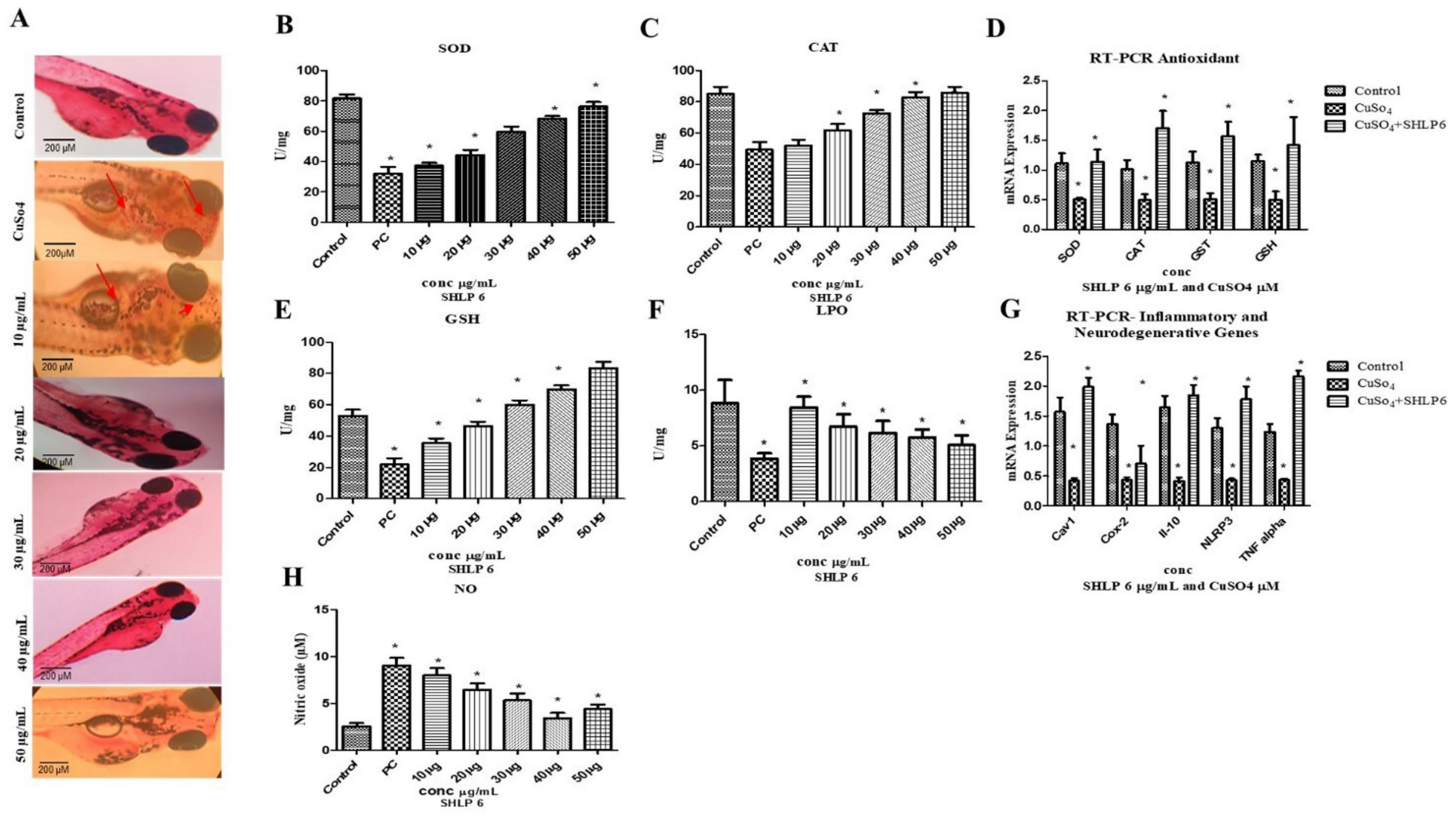
Macrophage accumulation, antioxidant enzyme activity, and gene expression analysis **(A)** Neutral red staining, **(B)** SOD, **(C)** CAT, **(D)** RT-PCR analysis of antioxidant genes, **(E)** GSH levels, **(F)** LPO levels, **(G)** RT-PCR analysis of proinflammatory and neurodegenerative genes, and **(H)** NO assay. Asterisks (*) indicate statistical significance at a *p*-value of <0.05.

Neutral red staining was used to observe macrophage migration in zebrafish larvae. Larvae exposed to CuSO_4_ showed the maximum macrophage migration, whereas SHLP6-treated larvae showed less macrophage accumulation, especially at 50 μg/mL (Figure 3A).

### Effects of SOD

In the CuSO_4_-exposed group, SOD levels were significantly decreased to 32 U/mg of protein compared to the control group, which had 81.6 U/mg of protein. SHLP6-treated larvae exhibited SOD levels of 37.3 U/mg, 44.2 U/mg, 59.5 U/mg, 68.3 U/mg, and 76.3 U/mg of protein, confirming its potential to enhance SOD levels in a concentration-dependent manner (Figure 3B).

### Effects of CAT

The CuSO_4_-exposed group exhibited lower CAT levels (49.4 U/mg of protein) compared to the control group (85 U/mg). In contrast, SHLP6 treatment increased the CAT levels to 52 U/mg, 61.8 U/mg, 72.6 U/mg, 82.9 U/mg, and 85.6 U/mg of protein. Therefore, SHLP6 improved the antioxidant defense against CuSO_4_-induced oxidative stress (Figure 3C).

### GSH activity

The CuSO_4_-exposed group had considerably lower GSH levels (20.8 U/mg of protein) than the control group (57.3 U/mg of protein). In SHLP6-treated larvae, GSH levels were significantly higher at 35.4 U/mg, 46.3 U/mg, 60 U/mg, 69.8 U/mg, and 83.3 U/mg of protein, and enzyme activity increased proportionally. SHLP6 treatment counteracted the CuSO_4_-induced oxidative stress (Figure 3E).

### LPO assay

The CuSO_4_-exposed stress group had significantly higher MDA levels (7.13 U/mg) than the control group (3.86 U/mg). In zebrafish larvae exposed to CuSO_4_, SHLP6 reduced the MDA levels by 8.4 U/mg, 6.7 U/mg, 6.18 U/mg, 5.26 U/mg, and 5.49 U/mg, respectively (Figure 3F).

### NO assay

NO levels were measured using the Griess reagent. Zebrafish larvae exposed to CuSO_4_ showed higher NO levels (9.04 μM) than the control group (2.5 μM). NO levels decreased in SHLP6-treated larvae (7.9 μM, 6.4 μM, 5.3 μM, 3.41 μM, and 4.4 μM) in a dose-dependent manner (Figure 3H).

### Gene expression analysis of inflammatory and neurodegenerative genes

RT-PCR analysis of antioxidant and inflammatory genes in CuSO_4_-induced zebrafish larvae revealed the downregulation of inflammatory genes (TNF-α [0.435-fold], NLRP3 [0.43-fold], Cox-2 [0.43-fold], and IL-10 [0.48-fold]) and the downregulation of antioxidant genes SOD (0.45-fold), CAT (0.46-fold), GST (0.50-fold), and GSH (0.509-fold). Compared with the control group, SHLP6 treatment upregulated the antioxidant genes SOD (1.3-fold), CAT (1.7-fold), GST (1.5-fold), and GSH (1.8-fold), while also upregulating the expression of the inflammatory markers Cox-2 (1.9-fold), TNF-alpha (2.1-fold), IL-6 (1.64-fold), and NLRP3 (1.41-fold). In addition to these effects, SHLP6 treatment also upregulated the expression of the anti-inflammatory marker IL-10 (1.73-fold). The 1.89-fold increase in Cav1 expression upon SHLP6 treatment suggests that the drug may enhance the function of caveolae, which play a significant role in signaling, endocytosis, and membrane stability. This increase indicates that the drug could be promoting or stabilizing caveolae-mediated signaling pathways, potentially impacting oxidative stress and inflammation (Figures 3D,G).

### Behavior analysis

**Figure 4:**
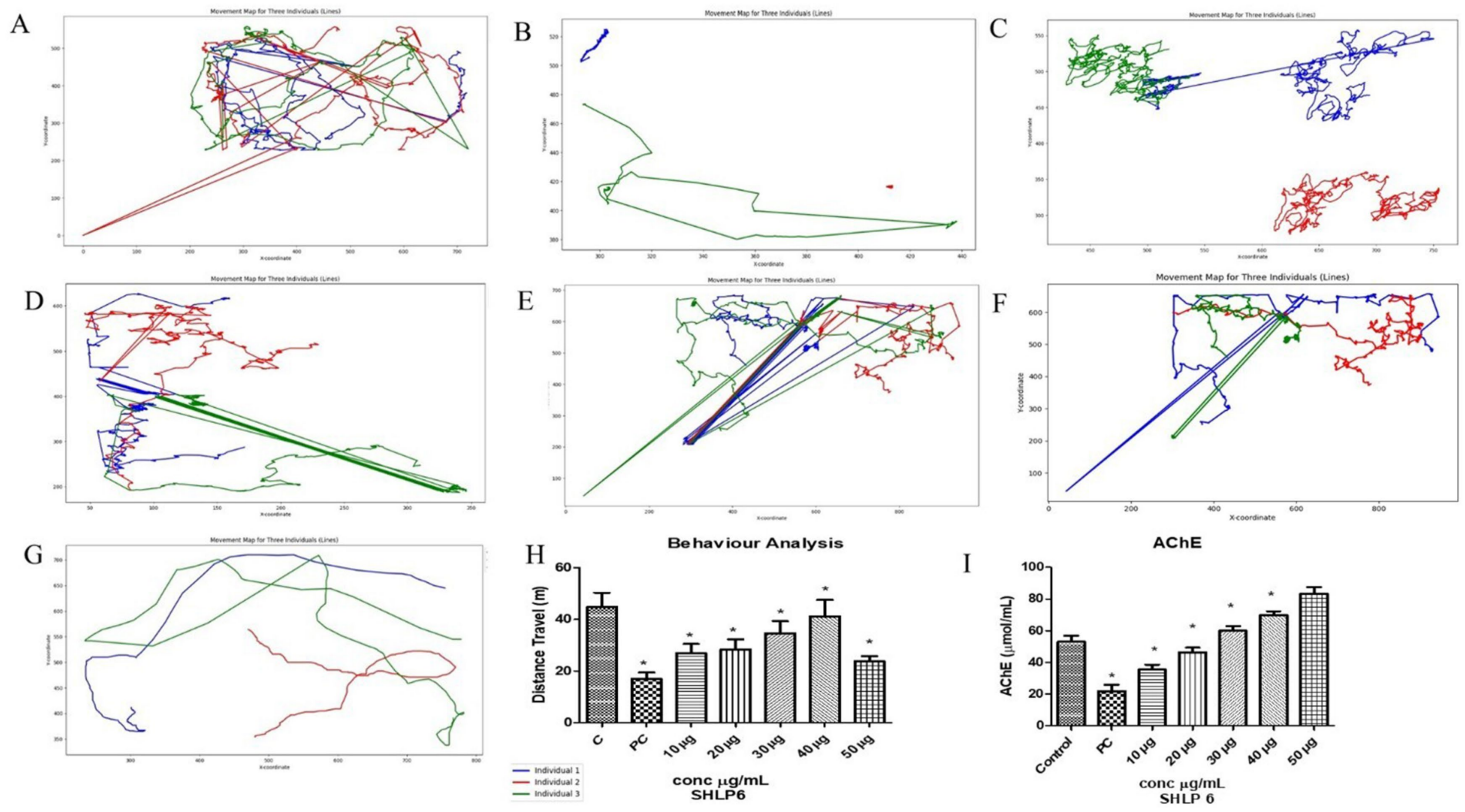
Behavior analysis. **(A)** Control, **(B)** CuSO_4_, **(C)** 10 μg/mL, **(D)** 20 μg/mL, **(E)** 30 μg/mL, **(F)** 40 μg/mL, **(G)** 50 μg/mL, **(H)** distance traveled, **(I)** AChE activity. Asterisks (*) indicate the statistical significance at a *p*-value of <0.05.

The locomotor study of zebrafish larvae was used to assess cognitive activity. The larvae exposed to CuSO_4_ swam at a shorter distance (16.76 m) than the control group (54.86 m). Treatment with SHLP6 improved swimming behavior at 26.9 m, 28.3 m, 34.6 m, 43.53 m, and 23.8 m compared to CuSO_4_-exposed larvae (Figures 4A–H).

### Acetylcholinesterase activity

AChE levels in zebrafish larvae exposed to CuSO_4_ were significantly lower (29.3 μmoL/mL) compared to the control group (82.3 μmoL/mL). However, zebrafish larvae treated with SHLP6 showed improved AChE levels by 39.7 μmoL/mL, 50.1 μmoL/mL, 57.2 μmoL/mL, 71.4 μmoL/mL, and 78.3 μmoL/mL in a concentration-dependent manner (Figure 4I).

## Discussion

Copper exposure induces oxidative stress by promoting excessive production of ROS, leading to LPO, DNA damage, and disruption of cellular functions (Vo et al., 2024). In the mitochondria, excess copper disrupts the function of the electron transport chain by interacting with respiratory complexes, resulting in electron leakage and the excessive production of ROS (Isei and Kamunde, 2020). This mitochondrial dysfunction leads to oxidative damage to mitochondrial DNA, lipids, and proteins, impairing energy production and initiating apoptosis (Zhou et al., 2024). This stress triggers inflammation and worsens cellular damage, thereby contributing to tissue dysfunction and disease progression (Sul and Ra, 2021). Several peptide therapeutics, such as melanocortin peptides, cortistatin, and adrenomedullin, have been used to reduce inflammation by modulating immune responses and decreasing the pro-inflammatory cytokine levels (Pintér et al., 2014; Benita and Koss, 2024). However, despite their effectiveness, some peptides may have minor side effects or lack target specificity, limiting their widespread therapeutic use.

Zebrafish larvae and embryos treated with SHLP6 peptide (10 to 50 μg/mL) exhibited a protective effect against CuSO_4_-induced malformations and toxicity. The SHLP6-treated group showed a better survival rate, confirming that the peptide can reduce the toxic effects of CuSO_4_. Treatment with SHLP6 (50 μg/mL) improved survival and heart rate compared to the untreated stress group. A study by Vijayanand et al. (2024) on the effect of the KC14 peptide from *Cyprinus carpio* against oxidative stress reported that H_2_O_2_-induced stress led to yolk sac enlargement in zebrafish larvae, whereas the KC14 peptide showed no malformation. Similarly, Singh et al. (2022) reported that CuSO_4_-induced oxidative stress lowered the survival rate of zebrafish larvae by 57%. In an *in vitro* antioxidant assay, SHLP6 showed a concentration-dependent effect, with the highest inhibition observed at 50 μg/mL of 58.7% in DPPH and 74.3% in ABTS, respectively. A study on the *in vitro* and *in silico* antioxidant activity of novel peptides by Wang et al. (2019) showed 98.5% scavenging activity in an *in vitro* antioxidant assay.

Fluorescent staining studies showed that SHLP6 reduced ROS, apoptosis, and LPO with fluorescence intensities of 20.3, 17.6, and 19.28%, respectively, *in vivo* in zebrafish larvae. Similarly, a study of RM12 peptide by Raju et al. (2021) reported that the peptide significantly reduced the ROS levels in the treatment group compared to the positive control. A study by Prabha et al. (2022) reported that RW20 peptide at 40 μM significantly reduced ROS and apoptosis levels by 39 and 43%, respectively. In Sarkar and Arockiaraj (2022) reported that TL15 peptide (80 μM) of sulfite reductase from spirulina reduced LPO levels in zebrafish larvae. Neutral red staining showed that SHLP6 reduced the accumulation of macrophages in zebrafish larvae compared to the untreated stress group. A study on spermidine against oxidative stress in zebrafish by Jeong et al. (2018) reported that spermidine significantly reduced the accumulation of macrophages.

Enzymatic assays proved the antioxidant properties of SHLP6 *in vivo* in zebrafish larvae. CuSO_4_ exposure drastically increased oxidative stress levels by triggering LPO and NO levels, which, in turn, suppressed the defense of antioxidant enzymes, including SOD and CAT, respectively. SHLP6 significantly reduced the MDA and NO levels by enhancing the levels of SOD, CAT, and GSH enzymes. Zhou et al. (2023) reported that CuSO_4_ diminished the first-line defense enzyme SOD levels. Similarly, GSH, which is also known as a detoxifying enzyme, showed its protective effect against CuSO_4_-induced stress *in vivo* in zebrafish larvae. A study by Prabha et al. (2022) reported similar results for RW20 against H_2_O_2_-stressed zebrafish larvae with a notable decrease in LPO and NO levels by improving the SOD, CAT, and GSH levels, respectively.

AChE levels and cognitive activity were evaluated. SHLP6 preserved AChE levels, whereas, in the untreated stress group, AChE levels markedly decreased, indicating the neuroprotective effect of SHLP6. In Prabha et al. (2022) reported a maximum AChE activity of RW20 (40 μM) at 6.3 μmoL/mL. Similarly, SHLP6 treatment prevented the cognitive activity in larvae exposed to CuSO_4_. In Sarasamma et al. (2019) reported that behavioral impairments are strongly associated with the central nervous system, which can be confirmed by the swimming patterns of zebrafish larvae.

Gene expression studies showed that SHLP6 significantly upregulated antioxidant genes, such as SOD, CAT, GST, and GSH. Similarly, the NV14 peptide upregulated the antioxidant enzymes that act as a first line of defense (Velayutham et al., 2021). Inflammatory markers such as TNF-α and NLRP3 were upregulated in the SHLP6-treated group. Although pro-inflammatory markers were highly expressed, the peptide upregulated the anti-inflammatory gene IL-10 as a counter-defense mechanism to regulate the inflammatory pathway. A study by Asai et al. (2024) reported that caveolin-1 peptide potentially inhibited inflammation. Therefore, SHLP6 can combat inflammation and oxidative stress by reducing ROS levels and modulating inflammatory mediators, such as TNF-α and IL-10.

These protective effects of SHLP-6 against CuSO_4_-induced toxicity may be mediated through key signaling pathways that regulate oxidative stress, inflammation, and mitochondrial function. One crucial pathway is Nrf2 signaling, which controls the antioxidant defense. Under oxidative stress, Nrf2 dissociates from its inhibitor Keap1 and translocates to the nucleus, where it binds to antioxidant response elements, upregulating detoxifying enzymes such as SOD, CAT, GPx, and GST (Ngo and Duennwald, 2022). SHLP-6 may activate Nrf2, leading to the upregulation of antioxidant enzymes by reducing oxidative damage. This activation enhances cellular resilience against ROS, reduces oxidative damage, and prevents mitochondrial dysfunction.

Additionally, SHLP-6 may modulate the MAPK signaling cascade and influence the ERK, JNK, and p38 pathways. While ERK supports cell survival, JNK, and p38 mediate stress responses and inflammation (Ki et al., 2013; Pua et al., 2022). By balancing these pathways, SHLP-6 may suppress pro-inflammatory cytokines (TNF-α, IL-1β), while enhancing anti-inflammatory IL-10 expression.

Furthermore, SHLP-6 may play a role in mitochondrial regulation, preserving membrane potential, reducing ROS production, and preventing cytochrome c release. By stabilizing mitochondrial dynamics, SHLP-6 may protect neurons from oxidative damage and apoptosis. The observed improvements in acetylcholinesterase activity and locomotor function in zebrafish suggest its neuroprotective potential. Although SHLP6 has potential antioxidant and anti-inflammatory properties, some limitations include enzymatic degradation, poor bioavailability, and a lack of targeted delivery, which may affect its therapeutic potential. Additionally, the potential immunogenicity and unintended side effects, compared to antioxidants like resveratrol and N-acetylcysteine, require further investigation. Future studies with higher experimental models are necessary to validate its efficacy, assess long-term safety, and elucidate the underlying mechanisms before considering SHLP-6 as a potential therapeutic candidate for neurodegenerative diseases.

## Conclusion

Metal-induced neurodegeneration has emerged as a critical area of research because of its pivotal role in the progression of neurological conditions. Dysregulation of transition metals like copper, iron, and zinc disrupts cellular homeostasis, leading to oxidative stress, protein aggregation, and neuronal damage, which are central to the pathogenesis of these diseases. The toxic effects of metals, particularly copper, are associated with the overproduction of ROS, mitochondrial dysfunction, and chronic inflammation.

This study highlights the therapeutic potential of SHLP6 as a multifaceted neuroprotective and antioxidant agent against CuSO_4_-induced toxicity. SHLP6 effectively mitigated the harmful effects of CuSO_4_ by enhancing cellular antioxidant defenses, reducing oxidative stress, and modulating neuroinflammatory pathways. SHLP6 upregulated the expression and activity of key antioxidant enzymes, such as SOD, CAT, GST, and GSH, which are critical in neutralizing ROS and maintaining cellular redox balance. The reduction in oxidative damage markers, such as LPO and NO levels, further emphasizes the peptide’s role in preventing oxidative injury at the cellular level. In addition to its antioxidant properties, SHLP6 showed significant neuroprotective effects. The peptide improved AChE activity and enhanced locomotor behavior in zebrafish larvae exposed to CuSO_4_, reflecting its ability to preserve neural function and cognitive activity. Furthermore, the anti-inflammatory properties of SHLP6 were evident through its modulation of pro-inflammatory and anti-inflammatory mediators. SHLP6 regulated the expression of both pro-inflammatory (TNF-α, NLRP3) and anti-inflammatory cytokines (IL-10), highlighting its potential in balancing inflammatory responses. The peptide downregulated the expression of TNF-α and NLRP3, while upregulating Cox-2 and IL-10, indicating its role in suppressing neuroinflammation and promoting anti-inflammatory responses. This treatment reduces neuroinflammation and supports cellular recovery. In addition, SHLP6 upregulated the expression of key antioxidant genes, such as SOD, CAT, GST, and GSH, thereby reinforcing the cellular antioxidant defense system. Improved acetylcholine esterase activity and restored locomotor performance further support its neuroprotective efficacy against CuSO_4_ toxicity. Its ability to upregulate genes associated with neuroinflammation and antioxidant pathways further underscores its protective effects, making it a promising candidate for treating metal-induced neurodegeneration.

Therefore, SHLP6 may play a pivotal role in addressing the pathophysiological mechanisms underlying neurodegenerative diseases associated with metal toxicity, such as Alzheimer’s disease, Parkinson’s disease, and other conditions driven by oxidative stress and inflammation. The peptide’s dual functionality, which is pro-apoptotic in cancer cells and protective in normal cells, makes it a versatile therapeutic agent. Future research may focus on clinical applications, exploring its pharmacokinetics, long-term safety, and synergistic effects with existing therapeutic approaches and higher experimental models, paving the way for SHLP6 to become a valuable tool in managing neurodegenerative diseases and oxidative stress-related conditions.

## Data Availability

The datasets presented in this study can be found in online repositories. The names of the repository/repositories and accession number(s) can be found in the article/supplementary material.

## Glossary

SHLP6: Small Humanin-Like Peptide-6
MDP: Mitochondrial Derived Peptide
sORF: Small Open Read Frame
DNA: Deoxy Ribonucleic Acid
mtDNA: Mitochondrial Deoxy Ribonucleic Acid
CuSO_4_: Copper sulfate
CAT: Catalase
SOD: Superoxide Dismutase
GST: Glutathione S-transferase
GPx: Glutathione Peroxidase
DPPH: 2,2-diphenyl-1-picrylhydrazyl
ABTS: 2,2′-azinobis(3-ethylbenzothiazoline-6-sulfonic acid)
DCFDA: 2′-7′-Dichlorodihydrofluorescein diacetate
AO: Acridine orange
DPPP: Diphenyl-1-pyrenylphosphine
TNF-α: Tumor Necrosis Factor Alpha
NLRP3: Nucleotide-binding domain, leucine-rich-containing family, pyrin domain–containing-3
COX-2: Cyclooxygenase-2
μg: Microgram
μm: Micromolar
YSE: Yolk sac edema
ROS: Reactive Oxygen Species
cm: Centimeter
mL: Milliliter

## References

[ref1] Asai C. Takamura N. Watanabe T. Asami M. Ikeda N. Reese C. F.. (2024). A water-soluble caveolin-1 peptide inhibits psoriasis-like skin inflammation by suppressing cytokine production and angiogenesis. Sci. Rep. 14:20553. doi: 10.1038/s41598-024-71350-1, PMID: 39232048 PMC11375059

[ref2] Benita B. A. Koss K. M. (2024). Peptide discovery across the spectrum of neuroinflammation; microglia and astrocyte phenotypical targeting, mediation, and mechanistic understanding. Front. Mol. Neurosci. 17:1443985. doi: 10.3389/fnmol.2024.1443985, PMID: 39634607 PMC11616451

[ref3] Brostjan C. Oehler R. (2020). The role of neutrophil death in chronic inflammation and cancer. Cell Death Discov. 6:26. doi: 10.1038/s41420-020-0255-6, PMID: 32351713 PMC7176663

[ref4] Chatterjee S. (2016). “Oxidative stress, inflammation, and disease” in Oxidative stress and biomaterials (Elsevier), 35–58. doi: 10.1016/B978-0-12-803269-5.00002-4

[ref5] Chen L. Deng H. Cui H. Fang J. Zuo Z. Deng J.. (2018). Inflammatory responses and inflammation-associated diseases in organs. Oncotarget 9, 7204–7218. doi: 10.18632/oncotarget.23208, PMID: 29467962 PMC5805548

[ref6] Focarelli F. Giachino A. Waldron K. J. (2022). Copper microenvironments in the human body define patterns of copper adaptation in pathogenic bacteria. PLoS Pathog. 18:e1010617. doi: 10.1371/journal.ppat.1010617, PMID: 35862345 PMC9302775

[ref7] Gao C. Jiang J. Tan Y. Chen S. (2023). Microglia in neurodegenerative diseases: mechanism and potential therapeutic targets. Signal Transduct. Target. Ther. 8:359. doi: 10.1038/s41392-023-01588-0, PMID: 37735487 PMC10514343

[ref8] Gordo A. C. Walker C. Armada B. Zhou D. (2017). Efficacy of celecoxib versus ibuprofen for the treatment of patients with osteoarthritis of the knee: a randomized double-blind, non-inferiority trial. J. Int. Med. Res. 45, 59–74. doi: 10.1177/0300060516673707, PMID: 28222627 PMC5536610

[ref9] Hernandez P. P. Undurraga C. Gallardo V. E. Mackenzie N. Allende M. L. Reyes A. E. (2011). Sublethal concentrations of waterborne copper induce cellular stress and cell death in zebrafish embryos and larvae. Biol. Res. 44, 7–15. doi: 10.4067/S0716-97602011000100002, PMID: 21720676

[ref10] Hohmann U. Ghadban C. Hohmann T. Kleine J. Schmidt M. Scheller C.. (2022). Nimodipine exerts time-dependent neuroprotective effect after excitotoxical damage in organotypic slice cultures. Int. J. Mol. Sci. 23:3331. doi: 10.3390/ijms23063331, PMID: 35328753 PMC8954806

[ref11] Isei M. O. Kamunde C. (2020). Effects of copper and temperature on heart mitochondrial hydrogen peroxide production. Free Radic. Biol. Med. 147, 114–128. doi: 10.1016/j.freeradbiomed.2019.12.006, PMID: 31825803

[ref12] Issac P. K. Guru A. Velayutham M. Pachaiappan R. Arasu M. V. Al-Dhabi N. A.. (2021). Oxidative stress induced antioxidant and neurotoxicity demonstrated in vivo zebrafish embryo or larval model and their normalization due to morin showing therapeutic implications. Life Sci. 283:119864. doi: 10.1016/j.lfs.2021.119864, PMID: 34358548

[ref13] Issac P. K. Velumani K. (2024). Rutin trihydrate conjugated zinc oxide nanoparticles targeting oxidative stress pathways for the protection of gut microbiome dysfunction and neurodegenerative diseases. BioNanoScience 14, 5310–5326. doi: 10.1007/s12668-024-01430-z

[ref14] Jeong J.-W. Cha H.-J. Han M. H. Hwang S. J. Lee D.-S. Yoo J. S.. (2018). Spermidine protects against oxidative stress in inflammation models using macrophages and zebrafish. Biomol. Ther. (Seoul) 26, 146–156. doi: 10.4062/biomolther.2016.272, PMID: 28365977 PMC5839493

[ref15] Jia B. Ye J. Gan L. Li R. Zhang M. Sun D.. (2022). Mitochondrial antioxidant SkQ1 decreases inflammation following hemorrhagic shock by protecting myocardial mitochondria. Front. Physiol. 13:1047909. doi: 10.3389/fphys.2022.1047909, PMID: 36467681 PMC9709459

[ref16] Ki Y.-W. Park J. H. Lee J. E. Shin I. C. Koh H. C. (2013). JNK and p38 MAPK regulate oxidative stress and the inflammatory response in chlorpyrifos-induced apoptosis. Toxicol. Lett. 218, 235–245. doi: 10.1016/j.toxlet.2013.02.003, PMID: 23416140

[ref17] Kuo C.-L. Lin Y.-C. Lo Y. K. Lu Y.-Z. Babuharisankar A. P. Lien H.-W.. (2024). The mitochondrial stress signaling tunes immunity from a view of systemic tumor microenvironment and ecosystem. iScience 27:110710. doi: 10.1016/j.isci.2024.110710, PMID: 39262792 PMC11388186

[ref18] Kwon H. S. Koh S.-H. (2020). Neuroinflammation in neurodegenerative disorders: the roles of microglia and astrocytes. Transl. Neurodegener. 9:42. doi: 10.1186/s40035-020-00221-2, PMID: 33239064 PMC7689983

[ref19] Lereim R. R. Nytrova P. Guldbrandsen A. Havrdova E. K. Myhr K.-M. Barsnes H.. (2024). Natalizumab promotes anti-inflammatory and repair effects in multiple sclerosis. PLoS One 19:e0300914. doi: 10.1371/journal.pone.0300914, PMID: 38527011 PMC10962820

[ref20] Li Y. Li Z. Ren Y. Lei Y. Yang S. Shi Y.. (2024). Mitochondrial-derived peptides in cardiovascular disease: novel insights and therapeutic opportunities. J. Adv. Res. 64, 99–115. doi: 10.1016/j.jare.2023.11.018, PMID: 38008175 PMC11464474

[ref21] López-Armada M. J. Riveiro-Naveira R. R. Vaamonde-García C. Valcárcel-Ares M. N. (2013). Mitochondrial dysfunction and the inflammatory response. Mitochondrion 13, 106–118. doi: 10.1016/j.mito.2013.01.003, PMID: 23333405

[ref22] Merry T. L. Chan A. Woodhead J. S. T. Reynolds J. C. Kumagai H. Kim S.-J.. (2020). Mitochondrial-derived peptides in energy metabolism. Am. J. Physiol. Endocrinol. Metab. 319, E659–E666. doi: 10.1152/ajpendo.00249.2020, PMID: 32776825 PMC7750512

[ref23] Messer J. S. (2017). The cellular autophagy/apoptosis checkpoint during inflammation. Cell. Mol. Life Sci. 74, 1281–1296. doi: 10.1007/s00018-016-2403-y, PMID: 27837217 PMC11107496

[ref24] Naoi M. Maruyama W. Shamoto-Nagai M. (2022). Neuroprotective function of rasagiline and selegiline, inhibitors of type B monoamine oxidase, and role of monoamine oxidases in synucleinopathies. Int. J. Mol. Sci. 23:11059. doi: 10.3390/ijms231911059, PMID: 36232361 PMC9570229

[ref25] Newsholme P. Cruzat V. F. Keane K. N. Carlessi R. de Bittencourt P. I. H. (2016). Molecular mechanisms of ROS production and oxidative stress in diabetes. Biochem. J. 473, 4527–4550. doi: 10.1042/BCJ20160503C, PMID: 27941030

[ref26] Ngo V. Duennwald M. L. (2022). Nrf2 and oxidative stress: a general overview of mechanisms and implications in human disease. Antioxidants 11:2345. doi: 10.3390/antiox11122345, PMID: 36552553 PMC9774434

[ref27] Pintér E. Pozsgai G. Hajna Z. Helyes Z. Szolcsányi J. (2014). Neuropeptide receptors as potential drug targets in the treatment of inflammatory conditions. Br. J. Clin. Pharmacol. 77, 5–20. doi: 10.1111/bcp.12097, PMID: 23432438 PMC3895342

[ref28] Prabha N. Guru A. Harikrishnan R. Gatasheh M. K. Hatamleh A. A. Juliet A.. (2022). Neuroprotective and antioxidant capability of RW20 peptide from histone acetyltransferases caused by oxidative stress-induced neurotoxicity in in vivo zebrafish larval model. J. King Saud Univ. Sci. 34:101861. doi: 10.1016/j.jksus.2022.101861

[ref29] Pua L. J. W. Mai C.-W. Chung F. F.-L. Khoo A. S.-B. Leong C.-O. Lim W.-M.. (2022). Functional roles of JNK and p38 MAPK signaling in nasopharyngeal carcinoma. Int. J. Mol. Sci. 23:1108. doi: 10.3390/ijms23031108, PMID: 35163030 PMC8834850

[ref30] Qamar N. John P. Bhatti A. (2020). Toxicological and anti-rheumatic potential of *Trachyspermum ammi* derived biogenic selenium nanoparticles in arthritic Balb/c mice. Int. J. Nanomedicine 15, 3497–3509. doi: 10.2147/IJN.S243718, PMID: 32547009 PMC7240025

[ref31] Raeeszadeh M. Saleh Hosseini S. M. Amiri A. A. (2022). Impact of co-administration of N-acetylcysteine and vitamin E on cyclophosphamide-induced ovarian toxicity in female rats. J. Toxicol. 2022, 1–7. doi: 10.1155/2022/9073405, PMID: 36051383 PMC9427260

[ref33] Raju S. V. Mukherjee A. Sarkar P. Issac P. K. Lite C. Paray B. A.. (2021). RM12 similar to substance P from tachykinin of freshwater murrel Channa striatus influence intracellular ROS in vitro fish erythrocytes and developmental toxicity and antioxidant enzymes in vivo zebrafish embryo. Fish Physiol. Biochem. 47, 1073–1085. doi: 10.1007/s10695-021-00950-9, PMID: 34021418 PMC8139370

[ref34] Rehman K. Akash M. S. H. (2016). Mechanisms of inflammatory responses and development of insulin resistance: how are they interlinked? J. Biomed. Sci. 23:87. doi: 10.1186/s12929-016-0303-y, PMID: 27912756 PMC5135788

[ref35] Sarasamma S. Audira G. Samikannu P. Juniardi S. Siregar P. Hao E.. (2019). Behavioral impairments and oxidative stress in the brain, muscle, and gill caused by chronic exposure of C70 nanoparticles on adult zebrafish. Int. J. Mol. Sci. 20:5795. doi: 10.3390/ijms20225795, PMID: 31752171 PMC6888079

[ref36] Sarkar P. Arockiaraj J. (2022). TL15 peptide of sulphite reductase from spirulina, arthrospira platensis exhibited anti-inflammatory and antioxidant defence role in CuSO_4_-stressed zebrafish embryo through pro-inflammatory cytokine and glutathione redox mechanism. Int. J. Pept. Res. Ther. 29:1. doi: 10.1007/s10989-022-10471-5

[ref37] Schulz K. Kerber S. Kelm M. (1999). Reevaluation of the Griess method for determining NO/NO−2 in aqueous and protein-containing samples. Nitric Oxide 3, 225–234. doi: 10.1006/niox.1999.0226, PMID: 10442854

[ref38] Shefrin A. E. Goldman R. D. (2009). Use of dexamethasone and prednisone in acute asthma exacerbations in pediatric patients. Can. Fam. Physician 55, 704–706, PMID: 19602654 PMC2718595

[ref39] Shi Y. Pulliam D. A. Liu Y. Hamilton R. T. Jernigan A. L. Bhattacharya A.. (2013). Reduced mitochondrial ROS, enhanced antioxidant defense, and distinct age-related changes in oxidative damage in muscles of long-lived *Peromyscus leucopus*. Am. J. Physiol. Regul. Integr. Comp. Physiol. 304, R343–R355. doi: 10.1152/ajpregu.00139.2012, PMID: 23325454 PMC4116415

[ref40] Singh M. Guru A. Sudhakaran G. Pachaiappan R. Mahboob S. Al-Ghanim K. A.. (2022). Copper sulfate induced toxicological impact on in-vivo zebrafish larval model protected due to acacetin via anti-inflammatory and glutathione redox mechanism. Comp. Biochem. Physiol. C Toxicol. Pharmacol. 262:109463. doi: 10.1016/j.cbpc.2022.109463, PMID: 36087706

[ref41] Su L.-J. Zhang J.-H. Gomez H. Murugan R. Hong X. Xu D.. (2019). Reactive oxygen species-induced lipid peroxidation in apoptosis, autophagy, and ferroptosis. Oxidative Med. Cell. Longev. 2019, 5080843–5080813. doi: 10.1155/2019/5080843, PMID: 31737171 PMC6815535

[ref42] Sudhakaran G. Prathap P. Guru A. Haridevamuthu B. Murugan R. Almutairi B. O.. (2022). Reverse pharmacology of Nimbin-N2 attenuates alcoholic liver injury and promotes the hepatoprotective dual role of improving lipid metabolism and downregulating the levels of inflammatory cytokines in zebrafish larval model. Mol. Cell. Biochem. 477, 2387–2401. doi: 10.1007/s11010-022-04448-7, PMID: 35575874

[ref43] Sul O.-J. Ra S. W. (2021). Quercetin prevents LPS-induced oxidative stress and inflammation by modulating NOX2/ROS/NF-kB in lung epithelial cells. Molecules 26:6949. doi: 10.3390/molecules26226949, PMID: 34834040 PMC8625571

[ref44] Velayutham M. Ojha B. Issac P. K. Lite C. Guru A. Pasupuleti M.. (2021). NV14 from serine O-acetyltransferase of cyanobacteria influences the antioxidant enzymes in vitro cells, gene expression against H_2_O_2_ and other responses in vivo zebrafish larval model. Cell Biol. Int. 45, 2331–2346. doi: 10.1002/cbin.11680, PMID: 34314086

[ref45] Velumani K. John A. Shaik M. R. Hussain S. A. Guru A. Issac P. K. (2024). Exploring sesquiterpene lactone as a dual therapeutic agent for diabetes and oxidative stress: insights into PI3K/AKT modulation. 3 Biotech. 14:205. doi: 10.1007/s13205-024-04050-2, PMID: 39170770 PMC11333395

[ref46] Vijayanand M. Issac P. K. Velayutham M. Shaik M. R. Hussain S. A. Guru A. (2024). Exploring the neuroprotective potential of KC14 peptide from *Cyprinus carpio* against oxidative stress-induced neurodegeneration by regulating antioxidant mechanism. Mol. Biol. Rep. 51:990. doi: 10.1007/s11033-024-09905-8, PMID: 39287730

[ref47] Vo T. T. T. Peng T.-Y. Nguyen T. H. Bui T. N. H. Wang C.-S. Lee W.-J.. (2024). The crosstalk between copper-induced oxidative stress and cuproptosis: a novel potential anticancer paradigm. Cell Commun. Signal 22:353. doi: 10.1186/s12964-024-01726-3, PMID: 38970072 PMC11225285

[ref48] Wang M. Li C. Li H. Wu Z. Chen B. Lei Y.. (2019). In vitro and in silico antioxidant activity of novel peptides prepared from Paeonia Ostii ‘Feng Dan’ hydrolysate. Antioxidants 8:433. doi: 10.3390/antiox8100433, PMID: 31581414 PMC6826969

[ref49] Wright H. L. Moots R. J. Bucknall R. C. Edwards S. W. (2010). Neutrophil function in inflammation and inflammatory diseases. Rheumatology 49, 1618–1631. doi: 10.1093/rheumatology/keq045, PMID: 20338884

[ref50] Zhang W. Xiao D. Mao Q. Xia H. (2023). Role of neuroinflammation in neurodegeneration development. Signal Transduct. Target. Ther. 8:267. doi: 10.1038/s41392-023-01486-5, PMID: 37433768 PMC10336149

[ref51] Zhang L. Yang Z. Yang M. Yang F. Wang G. Liu D.. (2022). Copper-induced oxidative stress, transcriptome changes, intestinal microbiota, and histopathology of common carp (*Cyprinus carpio*). Ecotoxicol. Environ. Saf. 246:114136. doi: 10.1016/j.ecoenv.2022.114136, PMID: 36242823

[ref52] Zhou H. Wu C. Jin Y. Wu O. Chen L. Guo Z.. (2024). Role of oxidative stress in mitochondrial dysfunction and their implications in intervertebral disc degeneration: mechanisms and therapeutic strategies. J. Orthop. Transl. 49, 181–206. doi: 10.1016/j.jot.2024.08.016, PMID: 39483126 PMC11526088

[ref53] Zhou S. Yang Q. Song Y. Cheng B. Ai X. (2023). Effect of copper Sulphate exposure on the oxidative stress, gill transcriptome and external microbiota of yellow catfish, *Pelteobagrus fulvidraco*. Antioxidants 12:1288. doi: 10.3390/antiox12061288, PMID: 37372018 PMC10295726

